# Race and Ethnic Differences in Diet Quality Across Older Adulthood

**DOI:** 10.1093/geroni/igaf122.1874

**Published:** 2025-12-31

**Authors:** Kyla Shea, Sarah Booth

**Affiliations:** Tufts University, Boston, Massachusetts, United States; Tufts University, Medford, Massachusetts, United States

## Abstract

Research on diet quality among different racial and ethnic groups across older adulthood is limited. This study aimed to examine diet quality in non-institutionalized U.S. adults aged 60-69 (n = 11,520), 70-79 (n = 7,202), and 80 + (n = 4,114) using NHANES data from 2005 to March 2020. Participants were categorized by race/ethnicity: non-Hispanic white (n = 11,792), non-Hispanic black (n = 5,038), Hispanic (n = 4,590), and ‘other’ (n = 1,416). The Healthy Eating Index 2015 (HEI) and component scores were calculated using the population ratio method, with results presented as weighted means (95% confidence intervals). Total HEI scores (possible range 0-100) were similar across race/ethnic groups in all age categories [range 61.9(60.5, 63.2) to 68.0(62.1, 73.8)]. However, significant differences in HEI components were observed. In all age groups, Non-Hispanic blacks had significantly lower scores for dairy [60-69: 3.6(3.4, 3.8); 70-79: 3.7(3.4, 4.0); 80+: 4.3(3.8, 4.8)], Hispanics had significantly lower scores for refined grains [60-69: 5.1(4.6, 5.6), 70-79: 5.0(4.4, 5.6), 80+: 4.8(3.89, 5.7)], and sodium scores were significantly lowest in those categorized as ‘other’ [60-69: 1.9(0.8, 3.0), 70-79: 2.5(1.4, 3.6), 80+: 1.2(0.2, 3.1)]. Non-Hispanic whites had significantly lower scores for greens and beans in the 70-79 and 80+ age groups [2.8(2.6, 3.1) and 2.1(1.9, 2.4), respectively]. Mean scores for total protein, seafood and plant protein, and whole fruit met the recommendations for all race and ethnic groups in all age groups. This study revealed racial and ethnic differences in HEI component scores despite similar overall diet quality, highlighting the need for culturally tailored interventions to improve diet quality in older adults.

